# Cyanobacterial Diversity and a New *Acaryochloris*-Like Symbiont from Bahamian Sea-Squirts

**DOI:** 10.1371/journal.pone.0023938

**Published:** 2011-08-22

**Authors:** Susanna López-Legentil, Bongkeun Song, Manel Bosch, Joseph R. Pawlik, Xavier Turon

**Affiliations:** 1 Department of Animal Biology (Invertebrates), University of Barcelona, Barcelona, Spain; 2 Center for Marine Science, University of North Carolina Wilmington, Wilmington, North Carolina, United States of America; 3 Scientific and Technical Services (Confocal Microscopy), University of Barcelona, Barcelona, Spain; 4 Center for Advanced Studies of Blanes, Spanish National Research Council, Blanes, Girona, Spain; Argonne National Laboratory, United States of America

## Abstract

Symbiotic interactions between ascidians (sea-squirts) and microbes are poorly understood. Here we characterized the cyanobacteria in the tissues of 8 distinct didemnid taxa from shallow-water marine habitats in the Bahamas Islands by sequencing a fragment of the cyanobacterial 16S rRNA gene and the entire 16S–23S rRNA internal transcribed spacer region (ITS) and by examining symbiont morphology with transmission electron (TEM) and confocal microscopy (CM). As described previously for other species, *Trididemnum* spp. mostly contained symbionts associated with the *Prochloron*-*Synechocystis* group. However, sequence analysis of the symbionts in *Lissoclinum* revealed two unique clades. The first contained a novel cyanobacterial clade, while the second clade was closely associated with *Acaryochloris marina*. CM revealed the presence of chlorophyll *d* (chl *d*) and phycobiliproteins (PBPs) within these symbiont cells, as is characteristic of *Acaryochloris* species. The presence of symbionts was also observed by TEM inside the tunic of both the adult and larvae of *L. fragile*, indicating vertical transmission to progeny. Based on molecular phylogenetic and microscopic analyses, *Candidatus Acaryochloris bahamiensis* nov. sp. is proposed for this symbiotic cyanobacterium. Our results support the hypothesis that photosymbiont communities in ascidians are structured by host phylogeny, but in some cases, also by sampling location.

## Introduction

Symbioses between cyanobacteria and marine invertebrates are common, especially in sponges (reviewed in [1]) and ascidians (reviewed in [2]). However, little is known about the nature of these symbioses and much remains to be described. In particular, few studies have employed molecular approaches to more accurately assess bacterial diversity and host-specificity [3], [4], [5], [6], [7], [8]. The majority of ascidian-microbe studies have focused on species within the ascidian family Didemnidae (Aplousobranchia), which often establish symbiotic relationships with unicellular cyanobacteria from the genera *Prochloron* (Prochlorales) and *Synechocystis* (Chroococcales). The type species of these genera are *Prochloron didemni*, first found in *Didemnum* spp. from Baja California [9], [10], and *Synechocystis trididemni*, found in the Caribbean ascidian *Trididemnum cyanophorum* [11]. The cell morphology of both cyanobacterial species is very similar [11], and molecular phylogenetic analyses revealed that they had evolved from a common cyanobacterial ancestor [12], [13].

More recently, a new oxygenic photoautotroph was found in the tropical didemnid *Lissoclinum patella* [14]. This photosymbiont was tentatively named *Acaryochloris marina* and presented a set of unique characteristics, the most remarkable being that it uses chlorophyll *d* (chl *d*) as a predominant photosynthetic pigment [14], [15]. Chl *d* is a minor photosynthetic pigment that was found in association with red macroalgae and though to be an artifact [16]. Since then, the presence of *Acaryochloris* has also been reported in other ascidian species. Kühl et al. [17] reported *Acaryochloris*-like cells growing on biofilms beneath the didemnid ascidians *L. patella, Diplosoma similis* and *D. virens*. Martínez-García et al. [8] also observed small patches of *Acaryochloris*-like cells on the basal tunic layer of the Mediterranean ascidian *Cystodytes dellechiajei* (Polycitoridae).

Although the role of photosymbionts in most symbiotic relationships is unknown, the few studies that have investigated ascidian-cyanobacterial symbioses proposed a mutualistic relationship (reviewed in [18] for *Prochloron* symbiosis), with direct transmission of symbionts between adult generations through the larva [19], [20], [21], [22], [23]. Vertical transmission allows the maintenance of the symbiotic relationship and ensures that offspring have immediate access to the microbes necessary for their survival [18], [24]. This strategy is believed to give hosts a competitive edge from an early stage, and it is normally associated with obligate symbioses.

In the Caribbean, the colonial ascidian *Trididemnum solidum* Van Name 1902 is known to overgrow and kill corals [25]. This species is distributed in patches and releases larvae throughout the year, the majority settling within 15 min [26]. Both the larvae and the adult of *T. solidum* are associated with cyanobacteria of the genus *Synechocystis* [12], [27], [28]. Concentrations of chl *a* are much higher in the larvae than in the adults, suggesting that the ascidian is highly dependent on its photosymbionts [28], [29]. *Trididemnum cyanophorum* Lafargue & Duclaux 1979 was first described together with its symbiont *Synechocystis trididemni*, which was present in high densities both in the tunic of the adult and of the larvae, suggesting an obligate symbiosis [11].

The cosmopolitan didemnid *Lissoclinum fragile* (Van Name, 1902) is also found in the Caribbean and is known to carry symbiotic cyanobacteria [30], [31]. Monniot [30] described the symbiont of *L. fragile* as an alga located in the cloacal cavities of the colonies, in the tunic pouches around the abdomen of each zooid, in the mantle surrounding the gonads, and in the surface layer of the larvae [30]. In contrast, Kott et al. [32] and Cox [33] reported that the symbiont of *L. fragile* was a species of *Prochloron*, usually found in patches on the surface of the colonies.

The aim of this study was to assess the diversity of the cyanobacterial community inhabiting didemnid ascidians from the Bahamas Islands. We established the genetic identity and diversity of both the ascidian hosts and their photosymbionts in order to better understand the degree of host-specificity. To achieve this goal, we determined host phylogeny by sequencing a fragment of the mitochondrial gene cytochrome oxidase I (COI) that is commonly used to determine species boundaries and diversity among ascidian taxa [34], [35], [36], [37], [38]. In order to identify and describe the photosymbiont diversity from within ascidian tissues, we sequenced a fragment of the 16S rRNA gene and the entire 16S–23S rRNA internal transcribed spacer region (16S–23S ITS). We also examined the morphology of the photosymbionts by transmission electron microscopy (TEM). Finally, we used confocal microscopy (CM) to investigate the presence of chlorophyll *d* (chl *d*) and phycobiliproteins (PBPs) in some of our ascidian samples.

## Materials and Methods

### Ascidian samples and identification

**Figure 1:**
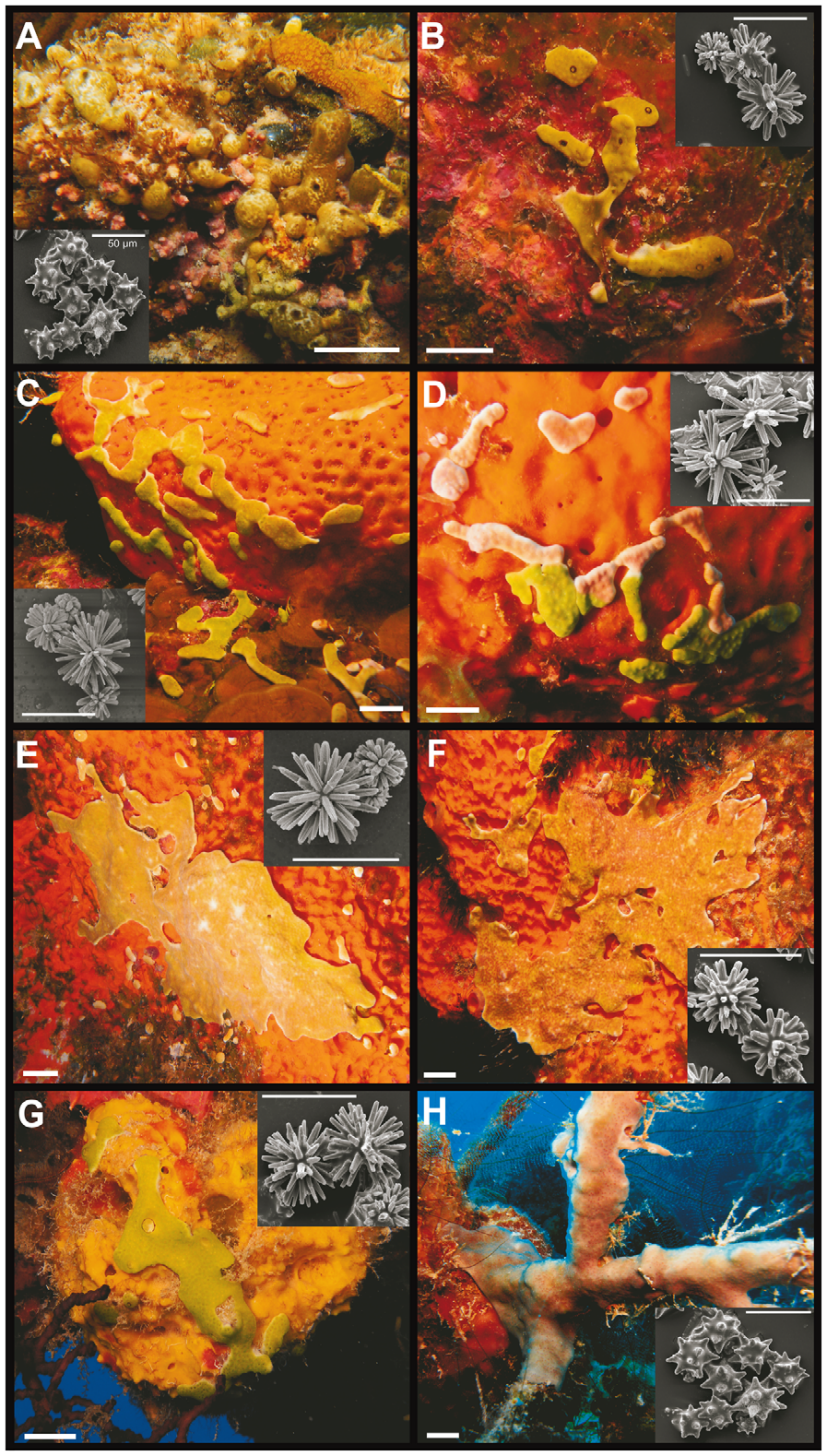
Images of Bahamian ascidians and their spicule types. (A) *Trididemnum cyanophorum* from Sweeting's Cay (SC 2-1); (B) *Lissoclinum* aff. *fragile* from Little San Salvador (LSS 1-7); (C) *Lissoclinum fragile* from West Plana Cay (WPC 1-1); (D) *Lissoclinum fragile* from West Plana Cay (WPC 3-6); (E) *Lissoclinum fragile* from East Plana Cay (EPC 1-2); (F) *Lissoclinum fragile* from East Plana Cay (EPC 1-5); (G) *Lissoclinum* aff. *fragile* from Little San Salvador (LSS 1-2); and (H) *Trididemnum solidum* from Little San Salvador (LSS 2-1). Scale bar on ascidian photos = 2 cm. Scale bar on spicule photos = 50 µm.

Ascidian samples were collected from mangroves of Sweeting's Cay, and coral reefs of Little San Salvador and Plana Cay (Bahamas) by SCUBA diving in 2008 and 2010 (Table 1). Collection of samples was performed with the permission of the Government of the Bahamas (to JRP). Pictures of each sampled colony were taken *in situ* before fixation in absolute ethanol (Figure 1). A piece of each colony was anaesthetized by cold exposure as described elsewhere [39], and fixed in formaldehyde for examination of zooids in a relaxed state. Spicules were obtained from small pieces of the tunic (≈5 mm^2^) previously boiled in commercial bleach until complete oxidation of the tissue. Spicules were then washed several times in water, dehydrated in absolute ethanol, and sputter-coated with gold. All spicule samples were observed with a Hitachi H1200 scanning electronic microscope available at the Scientific and Technical Services of the University of Barcelona (Figure 1). Samples were identified based on Lafargue & Duclaux [11], Kott [40], and Monniot [30], [41].

**Table 1:** Ascidian species analyzed in this study.

Species	Date	Code	Location	GPS position	Acc. Num. COI
*Trididemnum cyanophorum*	30-May-08	SC 2-1	Sweeting's Cay	26°38′35″N; 77°57′44″W	JF506187
*Lissoclinum* aff. *fragile* A	4-Jun-08	LSS 1-7	Little San Salvador	24°35′9″N; 75°58′26″W	JF506183
*Lissoclinum fragile* A	7-Jun-08	WPC 1-1	West Plana Cay	22°36′15″N; 73°37′39″W	JF506185
*Lissoclinum fragile* B	7-Jun-08	WPC 3-6	West Plana Cay	22°35′50″N; 73°37′45″W	JF506184
*Lissoclinum fragile* C	8-Jun-08	EPC 1-2	East Plana Cay	22°36′23″N; 73°33′37″W	JF506181
*Lissoclinum fragile* D	8-Jun-08	EPC 1-5	East Plana Cay	22°36′23″N; 73°33′37″W	JF506180
*Lissoclinum* aff. *fragile* B	9-Jun-08	LSS 1-2	Little San Salvador	24°35′7″N; 75°58′25″W	JF506182
*Trididemnum solidum*	9-Jun-08	LSS 2-1	Little San Salvador	24°35′6″N; 75°58′20″W	JF506186

Species name, sampling date, code, location within Bahamas, GPS position, and GenBank accession numbers for the cytochrome oxidase I (COI) gene.

All ascidians sampled for this study belonged to the family Didemnidae (Aplousobranchia). The sample collected in mangroves from Sweeting's Cay was identified as *Trididemnum cyanophorum*, while a similar species collected in Little San Salvador overgrowing corals was *T. solidum* (Figure 1, Table 1). The 4 samples collected on the reefs of East and West Plana Cay ranged in color from bright green to pink (i.e. Figure 1D), but examination of their zooid and spicule morphology revealed that they all belonged to the same species, identified as *Lissoclinum fragile* (Table 1). Samples collected on the pinnacles of Little San Salvador were morphologically similar to the ones collected in Plana Cay, although the pigmentation pattern was slightly different, with a black circle around the common cloacal apertures of the colonies. This feature was not described in the literature and was absent in other *L. fragile* colonies (Figure 1). To differentiate between these two groups of samples, we named the samples collected in Little San Salvador *Lissoclinum* aff. *fragile* (Table 1).

### Ascidian DNA extraction and PCR amplification

Samples fixed in absolute ethanol were kept at −20°C until processed. To maximize ascidian DNA yield, several zooids were carefully separated from the tunic under a stereomicroscope. DNA was extracted using the Puregene kit (Qiagen). The primer set LCO1490 and HCO2198 described by Folmer et al. [42], and Tun_forward and Tun_reverse2 described by Stefaniak et al. [43] were used to amplify a fragment of the Cytochrome *c* Oxidase subunit I (COI) mitochondrial gene. Amplification was performed with 1 µL of each primer (10 µM), 12.5 µL GoTaq™ reaction buffer (Promega), 10 to 20 µg/mL DNA, and PCR water to a total-reaction volume 25 µL. For both primer sets, a single soak at 94°C for 5 min, was followed by 40 amplification cycles (denaturation at 94°C for 30 sec; annealing at 40°C for 30 sec; and extension at 68°C for 2 min), and a final extension at 72°C for 10 min, in a Peltier PTC-200 gradient PCR. PCR products were purified using the Wizard purification kit (Promega). Sequencing reactions were carried out with the BigDye™ terminator v. 3.1 and the same primer set used during the amplification step, and analyzed on an ABI Prism 3100 automated sequencer. All sequences have been deposited in GenBank (accession numbers listed in Table 1).

### Cyanobacteria DNA extraction and PCR amplification

Several ascidian colonies per species and sampling site were ground together in absolute ethanol using a sterile pestle. The resulting greenish liquid was decanted into two 20 mL glass scintillation vials and evaporated under vacuum to leave a powdery organic residue. To re-suspend the extract, 1 mL of lysis solution from the Puregene kit (Qiagen) was added to each scintillation vial, vortexed, and incubated at 55°C for 65 min. The remaining solution was placed in two 2 mL sterile tubes and incubated for an additional hour at 55°C. After allowing the tubes to cool down, we continued with the DNA extraction protocol following manufacturer's instructions. The primer set CYA781F [44] and CYA23S1R or Primer 340 [45] was used to amplify the 3′ end of the 16S rRNA gene, the complete 16S–23S ITS region, and a small fragment of the 23S rRNA gene. Total reaction volume was 25 µL with: 12.5 µL of GoTaq mastermix (Promega), 1 µL of each primer (10 µM), 10.5 µL of PCR grade water, and 1 µL of DNA. PCR program was set as follows: A single soak at 85°C for 5 min, 38 amplification cycles (denaturation at 94°C for 1.5 min; annealing at 50°C for 2 min; and extension at 72°C for 3 min), and a final extension at 72°C for 10 min, in a Peltier PTC-200 gradient PCR. PCR products were run in a low-melting-point agarose gel (1%). One single band of around 1400 bp was observed for each sample, except for *Trididemnum cyanophorum* for which two clear bands were recorded (a ≈1400 bp band, hereafter named A; and a ≈1500 bp band, named B). Bands were processed separately and purified using the Wizard PCR Preps DNA Purification System (Promega).

Purified DNA was cloned in *E. coli* using the TOPO® TA Cloning® Kit and One Shot® TOP10 competent cells (Invitrogen), according to manufacturer's instructions. Eight separate clones per ascidian sample were screened by PCR using the plasmid primers T7 and M13R and a total reaction volume of 25 µL: 1 µL of each primer (10 µM), 12.5 µL of GoTaq mastermix (Promega), 10.5 µL of PCR grade water, and 1 µL of each clone, and the following cycle parameters: a single soak at 95°C for 10 min, followed by 30 amplification cycles (95°C for 30 s; 55°C for 30 s and 72°C for 1.5 min), and a final step at 72°C for 2 min. PCR amplicons were run in a low-melting-point agarose gel (1%) to confirm insert size before sequencing using BigDye TM terminator v. 3.1 on an ABI Prism 3100 automated sequencer. Because of the length of some of our sequences (up to 1445 bp), direct sequencing with primers T7 and M13R did not always result in a complete sequence, so we used the primer U1098F [46] to close any remaining gap. All sequences have been deposited in GenBank (accession numbers JF506188 to JF506255).

### Phylogenetic analysis

**Figure 2:**
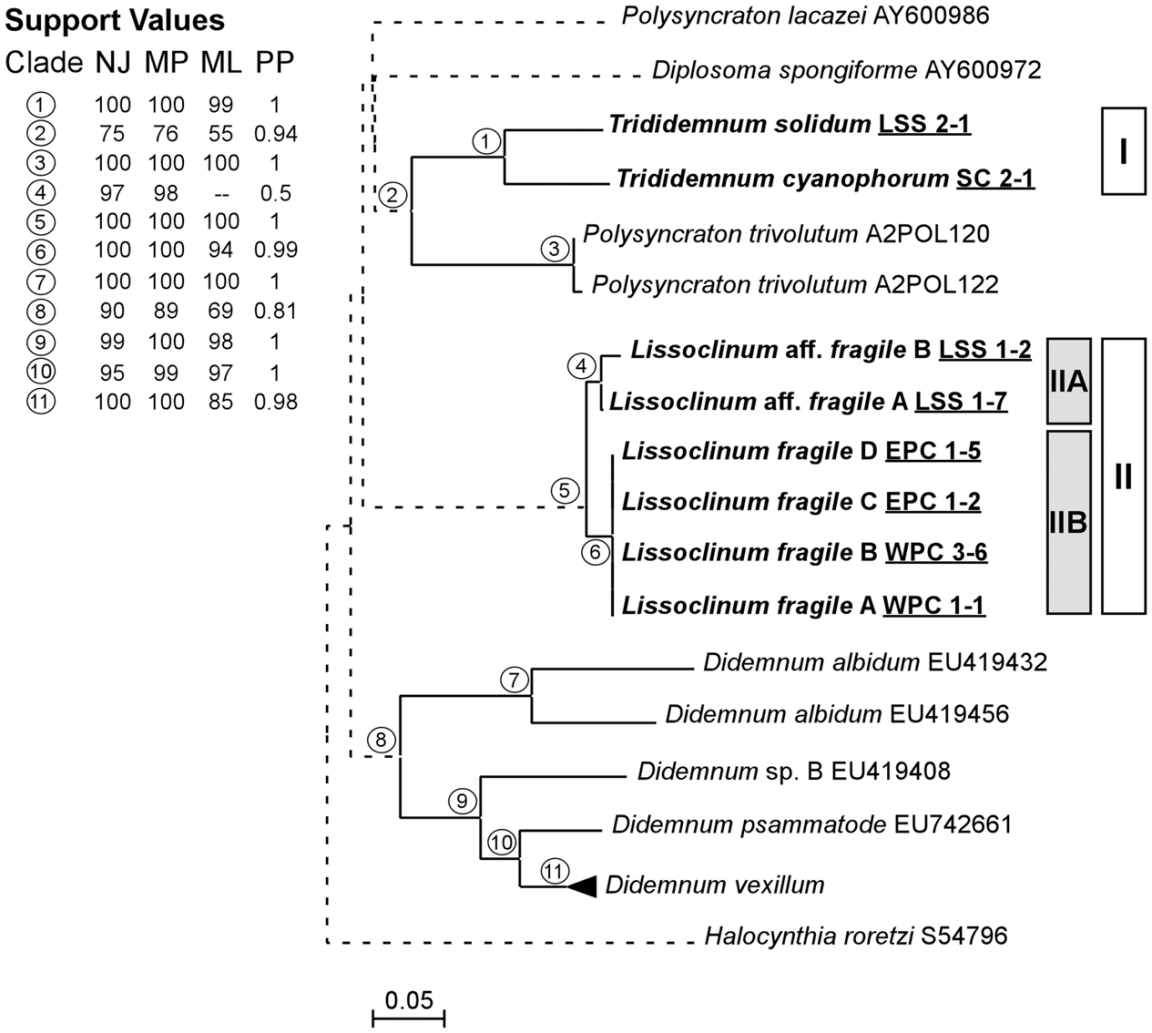
Host phylogeny with partial COI gene sequences. Sequences obtained in this study are highlighted (bold lettering). The Stolidobranchia species *Botryllus schlosseri* was used as an outgroup taxa. Labels on terminal nodes of reference sequences indicate the ascidian species and GenBank accession number or code in the Barcode of Life database. Labels on terminal nodes of sequences from this study include species name and sample code as in Table 1 (underlined: SC: Sweeting's Cay; LSS: Little San Salvador; WPC: West Plana Cay, and EPC: East Plana Cay). Bars and labels highlight the two clades of interest (I and II). Samples corresponding to *Lissoclinum* aff. *fragile* and *L. fragile* are further highlighted with a grey bar (IIA and IIB, respectively). Tree topology was obtained from neighbor-joining (NJ) analysis. Individual bootstrap values from NJ, maximum parsimony (MP) and maximum likelihood (ML) analyses and posterior probabilities (PP) from Bayesian inference are located in the upper-left box and correspond to circle numbers on tree nodes. Solid lines indicate bootstrap support greater than 50% from at least 2 of the 4 phylogenetic criteria, and dashed lines indicate weakly supported branches. The *Didemnum vexillum* cluster includes GenBank sequences: EU419439, -57, EU742661, -68, -69, -75. Scale bar represents 0.05 substitutions per site.

**Figure 3:**
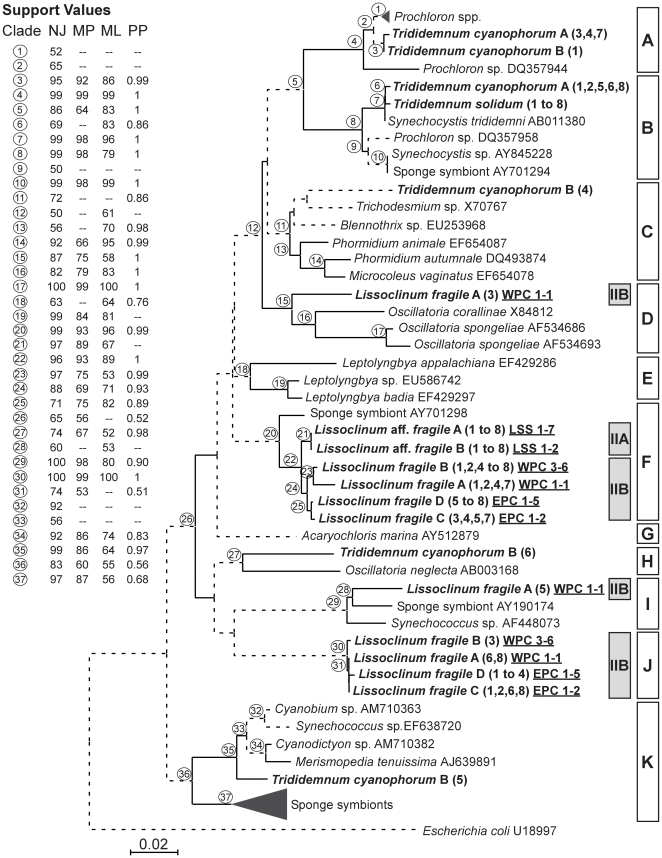
Phylogeny of partial 16S rRNA gene sequences from cyanobacteria isolated from Bahamian ascidians. Sequences obtained in this study are highlighted (bold lettering). Labels on terminal nodes of reference sequences indicate other cyanobacteria species and GenBank accession numbers. Labels on terminal nodes of sequences from this study include sample name, clone sequenced (in parenthesis), and sample code for the *Lissoclinum* species (underlined: LSS: Little San Salvador; WPC: West Plana Cay, and EPC: East Plana Cay). Tree topology was obtained from neighbor-joining (NJ) analysis. Individual bootstrap values from NJ, maximum parsimony (MP) and maximum likelihood (ML) analyses and posterior probabilities (PP) from Bayesian inference are located in the upper-left box and correspond to circle numbers on tree nodes. Solid lines indicate bootstrap support greater than 50% from at least 2 of the 4 phylogenetic criteria, and dashed lines indicate weakly supported branches. Bars and labels highlight the 11 major cyanobacterial groups detected in this study (A to K). Samples corresponding to *Lissoclinum* aff. *fragile* and *L. fragile* are further highlighted with a grey bar (IIA and IIB, respectively). The *Prochloron* spp. clade includes GenBank sequences: DQ357949, -50, -53, -57, -62, -66, -67, DQ385852, and X63141. The sponge symbiont clade includes GenBank sequences: AY701287, -1288, -1290, -1296, -1299, -1301, -1302, -1304, -1306, -1311, EU307448, -455, -458, -477, -488, -489, -492, -509, EF121775, -89, -98. Scale bar represents 0.02 substitutions per site.

**Figure 4:**
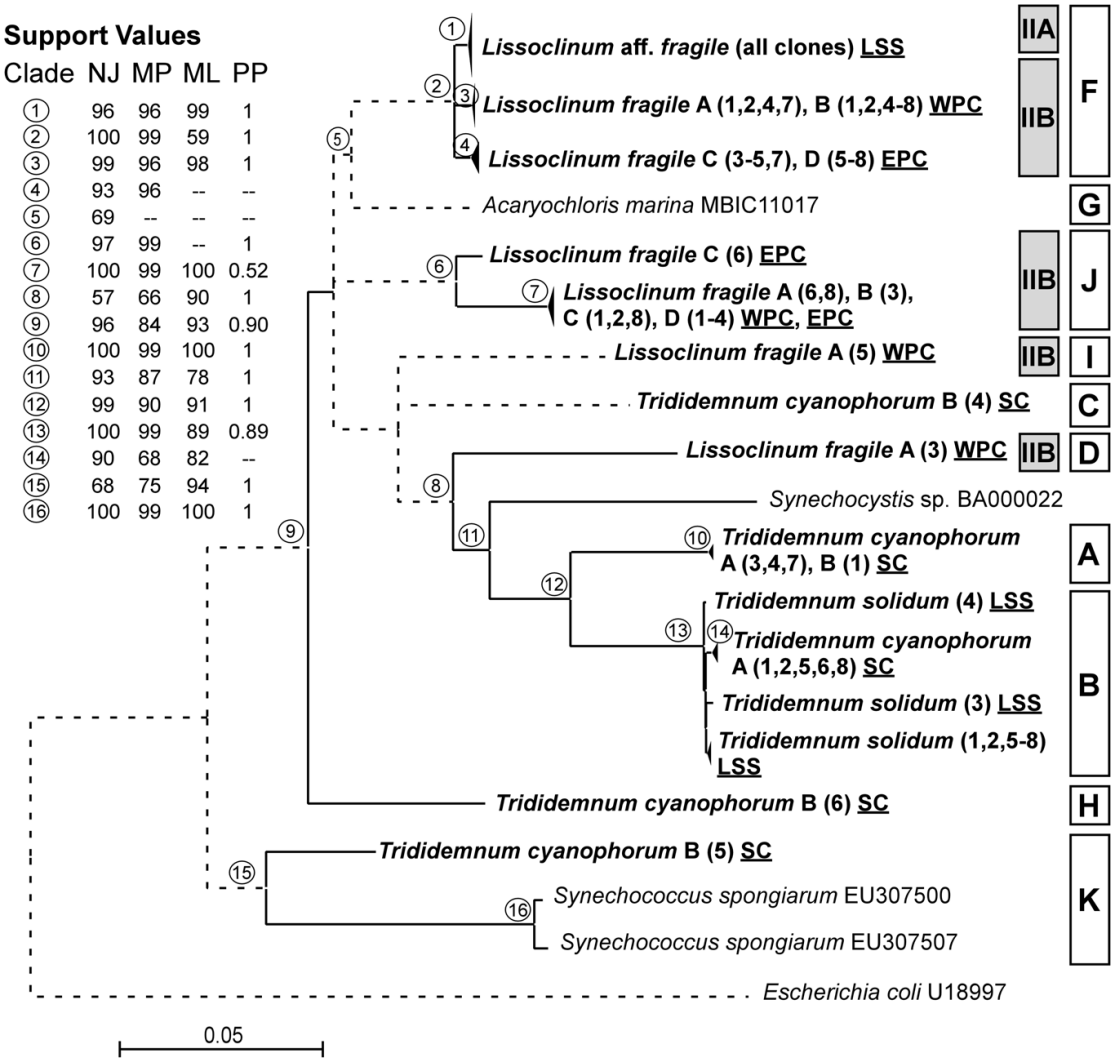
Phylogeny of 16S–23S ITS gene sequences from cyanobacteria isolated from Bahamian ascidians. Sequences obtained in this study are highlighted (bold lettering). Labels on terminal nodes of sequences include sample name and clone sequenced (in parenthesis). Labels on terminal nodes of sequences from this study include sample name, clone sequenced (in parenthesis), and sample location (underlined: SC: Sweeting's Cay, LSS: Little San Salvador; WPC: West Plana Cay, and EPC: East Plana Cay). Bars as in Figure 3. Tree topology corresponds to the consensus obtained from neighbor-joining (NJ) analysis. Individual bootstrap values from NJ, maximum parsimony (MP) and maximum likelihood (ML) analyses and posterior probabilities (PP) from Bayesian inference are located in the upper-left box and correspond to circle numbers on tree nodes. Solid lines indicate bootstrap support greater than 50% from at least 2 of the 4 phylogenetic criteria, and dashed lines indicate weakly supported branches. Scale bar represents 0.05 substitutions per site.

Sequences were aligned using Clustal X [47] with a gap opening penalty of 24 and a gap extension penalty of 4 [48]. To build each phylogenetic tree, additional sequences were retrieved from the Barcode of Life Data System [49] and GenBank (see accession numbers and codes in Figures 2, 3, and 4). Neighbor-joining (NJ) and maximum parsimony analyses were conducted in MEGA 4 [50]. For NJ analyses, the Tajima-Nei model of nucleotide substitution was used and data were re-sampled using 10,000 bootstrap replicates. For MP analyses, the Close-Neighbor-Interchange (CNI) branch-swapping algorithm was implemented and data were re-sampled using 1,000 bootstrap replicates [51]. JModeltest [52] was used to select the best model of DNA substitution for maximum likelihood (ML) and Bayesian inference (BI) analyses according to the Akaike information criterion (AIC). To analyze the COI and 16S rRNA sequences, the GTR+I+G [53] model with substitution rates varying among sites according to an invariant and gamma distribution was used. For sequences including the ITS region, we used the F81+G model [54] with substitution rates varying among sites. ML analyses were performed with Treefinder v. October 2008 [55] and the GTR+I+G substitution model. Data were re-sampled using 1000 bootstrap replicates. For Bayesian inference, MrBayes 3.1.2 [56] was run to obtain a majority-rule consensus tree and posterior probabilities of branch nodes, implementing the corresponding likelihood model for each gene fragment. To analyze the 16S rRNA gene fragment, and the same fragment plus the full ITS region, the Monte Carlo Markov Chain length was set to 16 and 4 million generations, respectively, with sampling every 100^th^ generation. After 15,013,000 generations (16S rRNA fragment) and 3,550,000 (16S–23S ITS region), the average standard deviation of split frequencies between two independent chains reached less than 0.01. For the ascidian sequences, the Monte Carlo Markov Chain length was set to 1,000,000 with sampling every 100^th^ generation. The average standard deviation of split frequencies between two independent chains reached less than 0.01 after 404,000 generations. For all analyses, the first 25% of the resulting trees were discarded as burn-in.

### Transmission electron microscopy

The ultrastructure of the most common symbionts in the ascidian tunic was examined by transmission electron microscopy (TEM). Small pieces (about 2 mm^3^) of *Lissoclinum fragile*, *Lissoclinum* aff. *fragile*, *Trididemnum cyanophorum*, and *T. solidum* were cut from each colony, and fixed in 2.5% glutaraldehyde 2% paraformaldehyde using filtered seawater as buffer. Only samples of *L. fragile* contained mature larvae, which were carefully separated from adults and observed separately. Samples were incubated in the fixative mixture overnight at 4°C, and then washed several times in filtered seawater. To construct resin blocks, samples were dehydrated in a graded ethanol series and embedded in Spurr's resin at room temperature. Semi-thin (5 microns) and ultrathin sections (ca. 60 nm) were cut with a Reichert Ultracut microtome. Ultrathin sections were stained with uranyl acetate and lead citrate for ultrastructural observation [57]. TEM observations were performed at the Microscopy Unit of the Scientific and Technical Services of the University of Barcelona on a JEOL JEM-1010 (Tokyo, Japan) coupled with a Bioscan 972 camera (Gatan, Germany).

### Confocal microscopy

Based on plylogenetic results, further analyses were performed to assess whether chlorophyll *d* (chl *d*) and phycobiliproteins (PBPs) were present in the tunic of *Lissoclinum* aff. *fragile* using confocal microscopy. Slices of two colonies previously fixed in 4% formaldehyde were mounted on a slide with distilled water. Images were acquired on a Leica TCS SPE confocal microscope equipped with a NPLAN 100× 1.2 water immersion objective. A 635 nm solid state laser was used for fluorescence excitation. Detection ranges for chl *d* and PBPs were set to 700–750 nm and 640–670 nm, respectively. Laser power and PMT gain and offset were equally set up for both signals. Confocal images were acquired at the maximum resolution of the objective and the stacks were deconvolved with Huygens Essential (Scientific Volume Imaging, B.V.). Deconvolution was calculated with the optimized iteration mode of this software and all image parameters were read from the Leica. lif format. Object counting and measurements were performed on the deconvolved image stacks after 3D surface rendering using Imaris (Bitplane A.G.) and the Imaris MeasurementPro module. Length and width of at least 80 cells per sample was calculated using the software ImageJ 1.41o. Each stack was analyzed setting the fluorescence threshold to the value that resulted in the maximum number of individual objects identified. Quantification was performed on those objects that showed both signals (PBPs and chl *d*). Final artwork was done with Photoshop CS (Adobe). Chl *d* and PBPs images were colored in red and green, respectively.

## Results and Discussion

### Host phylogeny

Partial COI gene sequences were obtained for all the samples, with a final alignment length of 605 bp. As expected, phylogenetic analyses grouped the two *Trididemnum* sequences studied in a well-supported clade (bootstrap values >99% in all analysis; Figure 2I). The taxonomic status of *T. cyanophorum* has been argued in the past, with some authors suggesting that it may be a synonym of *T. solidum* [30]. Here, we have found 12% sequence variability between *T. solidum* and *T. cyanophorum* and some morphological differences in terms of color and colony shape (Figure 1), suggesting that both species names are valid despite the lack of morphological differences among zooids. All the sequences of *Lissoclinum* obtained in this study formed a well-supported clade (bootstrap values = 100% in all analysis; Figure 2II). Moreover, within the *Lissoclinum* clade, two subgroups with 3% sequence variability were further distinguished. The first subgroup grouped the two samples obtained from Little San Salvador and named *Lissoclinum* aff. *fragile* (Figure 2 IIA). The second subgroup was formed by identical sequences for *L. fragile* from Plana Cay (Figure 2 IIB). All in all, phylogenetic analyses confirmed morphological observations and determined the taxonomic status of species and groups of samples presenting small morphological differences.

### Cyanobacteria phylogeny

After alignment, 68 partial 16S rRNA gene sequences ranging between 740 and 756 bp were obtained, 19 of which were unique. All sequences from *Lissoclinum* aff. *fragile* were identical and differed from the ones obtained for *L*. aff, *fragile* B by one mutation. Ten unique sequences were retrieved from *L. fragile*, 3 of which were only encountered in one clone. All sequences obtained from *Trididemnum solidum* were identical, while 6 unique sequences were retrieved from *T. cyanophorum*, 4 of which were only sequenced once. The same sequence was never found in both *Lissoclinum* and *Trididemnum* species. Analyses conducted using the 16S rRNA fragment described above plus the complete ITS region (269–647 bp) plus a small fragment of the 23S rRNA gene (44 bp), hereafter called the16S–23S ITS region, yielded 56 unique sequences. Identical sequences were observed among specimens of *L*. aff. *fragile* A and B (6 clones total), and within clones of *L*. aff. *fragile* B (2 clones), *L. fragile* B (2 clones) and *T. cyanophorum* A (2 clones).

Blast searches in GenBank with the 16S rRNA fragment showed that the best match for *Trididemnum solidum* and *Trididemnum cyanophorum* symbionts were uncultured *Prochloron* and *Synechocystis* sequences (>97% max. identity; >93% coverage in all cases). Three other sequences obtained for *T. cyanophorum* B (clones 4, 5, 6) shared similarities with a range of uncultured cyanobacteria, including Oscillatoriales and Chroococcales. All the sequences from *Lissoclinum* aff. *fragile* and most of the sequences from *L. fragile* (19) shared up to 95% identity with *Acaryochloris* sp. (100% coverage). The remaining sequences from *L. fragile* (11 in total) moderately resembled *Leptolyngbia antactica* (93% max. identity; 100% coverage). Finally, two sequences of *L. fragile* from West Plana Cay (A, clones 3 and 5) shared 94% and 97% identities with *Phormidium* sp. (100% coverage) and *Synechococcus* sp. sequences (100% coverage), respectively.

Phylogenetic analyses based on a fragment of the 16S rRNA gene (Figure 3), and the 16S–23S ITS region (Figure 4), revealed a total of 11 groups, 10 of which were equivalent among analyses. The first one (named A in Figure 3 and 4) included 4 sequences obtained from *T. cyanophorum* and had strong bootstrap support (>99%). Phylogenetic analyses conducted with the 16S rRNA fragment showed that these sequences from *T. cyanophorum* grouped with 9 sequences of *Prochloron* spp. retrieved from GenBank. Five sequences from *T. cyanophorum* and all sequences from *T. solidum* (8) were grouped with two species of *Synechocystis* and one sequence of *Prochloron* in clade B (Figure 3 and 4). High similarity of the cyanobacterial sequences obtained here for *T. solidum* and *T. cyanophorum* with *Synechocystis trididemni* is not surprising (clade B), as this cyanobacterium was first described in *T. cyanophorum* [11], and has also been found in other *Trididemnum* species [3]. Along with *Synechocystis*, some *Trididemnun* species can also establish symbiotic relationships with *Prochloron* (e.g. *T. miniatum* [58], *T. paracyclops* [3]). For *T. cyanophorum*, symbionts of both *Prochloron* and *Synechocystis* appeared to coexist. This phenomenon has also been reported for other ascidians, and is especially common in *Trididemnum* species [32], [59], [60].

The third clade (named C) from the 16S rRNA analyses was formed by different cyanobacterial species belonging to the Chroococcales, together with one sequence obtained here for *T. cyanophorum* B (clone 4). The position of the sequence from *T. cyanophorum* B (4) within this group was not resolved. Further analyses, including those of the 16S–23S ITS region, did not yield clearer results, probably due to the limited number of available cyanobacterial ITS sequences in GenBank (Figure 4). Other sequences of Chroococcales were found in the 16S rRNA clade I, which grouped clone 5 from *L. fragile* A, an uncultured cyanobacterium sequence obtained from the Australian sponge *Cymbastela* sp., and a cultured strain of *Synechococcus* sp. This clade had a bootstrap support >80% in all analyses, however, it did not form a monophyletic group with other *Synechococcus* sequences retrieved from GenBank (located in clade K). Clade K was entirely formed by Chroococcales, including many sponge symbionts, some free-living species, and clone sequence 5 from *T. cyanophorum* B (Figure 3 and 4). Cyanobacteria are common members of sponge-associated microbial communities. In particular, *Synechococcus* species have been widely reported as the major photosymbionts inhabiting sponges [48], [61], [62], [63]. Some of these symbionts were reported to be host-specific, others were found in different sponge species, and some varied according to location [48]. The relative integration of these symbionts varies depending on the sponge species, ranging from obligate symbiosis to a commensal existence, with cyanobacterial cells interspersed among sponge cells [64], [65]. In this study, only one sequence related to the sponge symbiont *Synechococcus* was found, indicating that this cyanobacterial genus may only form facultative associations in ascidians. Moreover, the sequence obtained herein appeared more closely related to free-living *Synechococcus*, and may just have been captured from the water column by the ascidian.

In the 16S rRNA analyses, one sequence (clone 5) obtained from *L. fragile* A formed clade D with cyanobacterial species belonging to the Oscillatoriales (Figure 3). In contrast, the 16S–23S ITS sequence obtained for *L. fragile* A (5) (Figure 4) held a basal position within the *Prochloron-Synechocystis* group (clades A and B). Other sequences of Oscillatoriales appeared in clade H, which, for the 16S rRNA analysis, only included 2 sequences: one obtained from *T. cyanophorum* B (clone 6) and a sequence from *Oscillatoria neglecta*. Although the existence of this clade appeared to be supported by bootstrap analysis, its connection with the other clades remained undetermined, especially considering that other Oscillatoriales were grouped in clade D (Figure 3). The 16S–23S ITS analysis (Figure 4) showed that the *T. cyanophorum* B (clone 6) sequence appeared basal to all clades except clade K (bootstrap support >84% in all analyses). Thus, based on 16S rRNA and 16S–23S ITS results, the identity and phylogenetic position of *L. fragile* A (clone 5) and *T. cyanophorum* B (clone 6) within the Oscillatoriales could not be resolved.

The last clade formed by sequences of Oscillatoriales was found only in the 16S rRNA analysis and grouped three sequences of *Leptolyngbya* spp. retrieved from GenBank (clade E, Figure 3). Although Blast searches returned *Leptolyngbya* species as best match for 11 of our 16S rRNA sequences from *L. fragile*, none of them were included in this clade based on phylogenetic analyses. Several studies have reported the occurrence of Oscillatoriales associated with ascidians [9], [33], [66], [67], [68], [69], [70], [71]. However, most of these studies include only short references to these symbionts [9], [67], [68], a few are accompanied by electron microscopical observations [33], [66], [69], [70], [71], and even fewer provided a name or a sequence to identify the symbiont [66], [70]. Thus, to date, the degree of host-specificity and strength of association between ascidians and Oscillatoriales remains unresolved.

The 16S rRNA clade F (Figure 3) grouped most of the sequences obtained from *L. fragile* A, B, C, and D, all the sequences from *L*. aff. *fragile*, and a single sequence obtained from the Bahamian sponge *Pseudoaxinella flava* by [63]. The best matches of this group according to blast searches were *Acaryochloris* species. Based on 16S rRNA analyses, *Acaryohcloris marina* (clade G in Figure 3) appeared at the base of clades A, B, C, D, E and F, while the 16S–23S ITS analyses showed that *A. marina* was related only to clade F (Figure 4). *Acaryochloris marina* is a recently discovered oxygenic photoautotroph that uses chl *d* as the predominant photosynthetic pigment [17], [72], [73]. A closer analysis of the *Acaryochloris*-like sequences (clade F) compared to *A. marina* (clade G) reported a sequence divergence >5%, while sequence variation within the *Acaryochloris*-like clade obtained here was <1%.

Within clade F, sequences were grouped according to host and sampling location. The first sub-clade contained all sequences of *L*. aff. *fragile* from Little San Salvador (Figures 3 and 4, IIA). Sequences for *L. fragile* formed two groups according to sampling site: West Plana Cay (samples A and B), and East Plana Cay (samples C and D, 7 kilometers away from West Plana Cay, and 330 km away from Little San Salvador). Thus, even adjacent populations had distinct cyanobacterial symbionts. All the remaining sequences of *L. fragile* A, B, C, and D formed a well-supported clade (J) by themselves, without any clear correlation with location (bootstrap support >97% in all analyses except ML for 16S–23S ITS; Figure 3 and 4). Therefore, as previously reported for sponges [48], some ascidian photosymbionts are host-specific, while a few may depend on environmental parameters associated with a given location.

The phylogenetic position of clade J within the cyanobacteria could not be resolved with analyses of the 16S rRNA fragment or the 16S–23S ITS region. Moreover, although the closest blast match with an identified cyanobacterium was *Leptolyngbia antactica*, none of the phylogenetic analyses related clade J with other Oscillatoriales (clades D, E, and H; Figure 3 and 4). Based on currently available information, we are unable to identify or provide a taxonomic position for sequences in clade J.

### Cyanobacteria morphology

**Figure 5:**
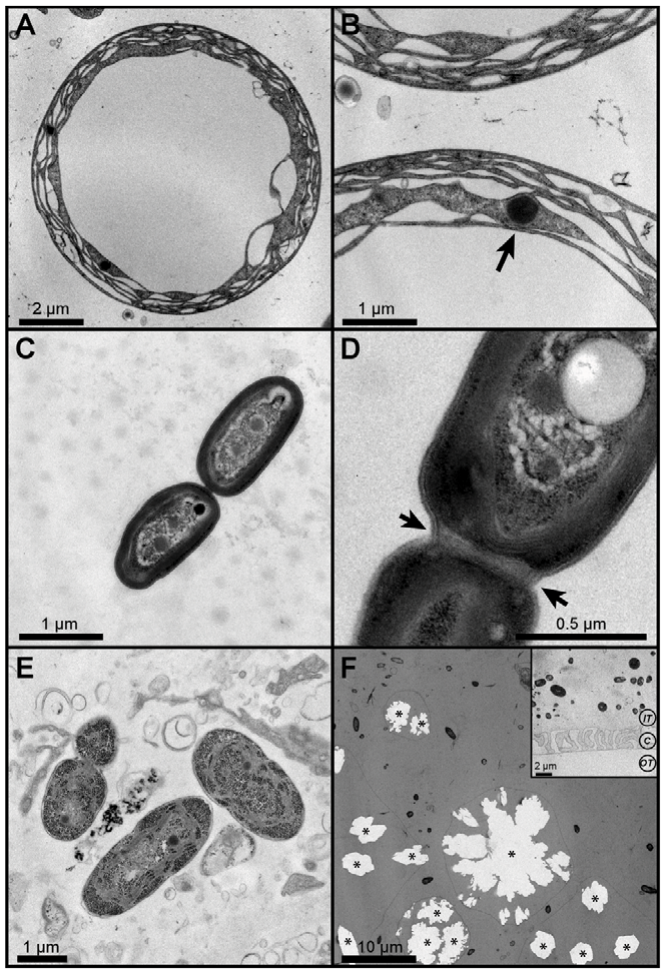
Transmission electron microscopical images of photosymbionts from *Trididemnum cyanophorum* and *Lissoclinum* aff. *fragile*. (A) *Synechocystis* sp. symbiont in *Trididemnum cyanophorum*; (B) Polyedric bodies located in the periphery of the *Synechocystis* cell; (C) *Acaryochloris bahamiensis* nv. sp. after division in the tunic of *Lissoclinum fragile*; (D) Detail showing the peptoglycan layer between two cells of *Acaryochloris bahamiensis* nv. sp. during the final stage of division; (E) Other cyanobacterial symbionts observed in the tunic of *L. fragile*; (F) Spicules and cyanobacteria distribution inside the tunic of *L. fragile* (asterisks indicate spicule location). The inset shows cyanobacteria in the tunic of the larvae (C: Cuticle; IT: Inner tunic; OT: Outer tunic).

Photosymbionts of *Trididemnum solidum* and *T. cyanophorum* were embedded in the adult tunic, but were never observed to be in direct contact with ascidian cells (Figure 5A). The symbionts observed in *T. solidum* and *T. cyanophorum* symbionts were morphologically identical to those reported from previous studies of *Synechocystis trididemni* [11]. Specifically, *S. trididemni* was reported to be round, with a diameter of 8 to 11 µm, and 5 to 7 thylakoids around the periphery of the cell. The diameter of *Synechocystis* observed herein fell within the range indicated above, however they had a few less thylakoids per cell (3 to 5 instead of 5 to 7). As described for *S. trididemni*, some of these thylakoids were also irregular and developed bladders or vesicles close to the nucleoplasma [11]. Polyedric bodies were also observed in close contact with the thylacoides (Figure 5B, [11]). Thus, morphological observations of the ultrastructure of photosymbionts of *T. solidum* and *T. cyanophorum* confirmed our phylogenetic results, indicating that *Synechocystis* was the major symbiont in these ascidian species.

A very different type of cyanobacterium was observed in *L. fragile* and *L*. aff. *fragile* samples (Figure 5). As hinted by the phylogenetic analyses, the overall structure of the photosymbionts (i.e. cell shape, size, and major features) was consistent with previous research describing the ultrastructure of *Acaryochloris* species [14], [74], [75], [76]. A consensus on the number and arrangement of the thylakoids within *Acaryochloris* cells does not exist in the literature. The first studies on *A. marina* reported more than 7 thylakoids surrounding the cytoplasm [14], [74], [75], while subsequent studies reported a lower number of thylakoids evenly spaced along the periphery of the cell [76], [77]. The *Acaryochloris*-like cells observed herein better fit this last description, and presented 5 to 7 thylakoids evenly spaced along the periphery of the cell (Figure 5C). Several symbiont cells were also observed undergoing division, and followed the major division steps described by Marquart et al. [75]. Notably, the photosymbiont cells observed herein also remained connected by a common peptidoglycan layer after cell division (Figure 5D). Another cyanobacterial type containing abundant glycogen granules was observed in the tunic around the zooid abdomen (Figure 5E). Cyanobacteria were consistently found outside the sheaths that surround the calcareous spicules of the tunic (Figure 5F).

*Acaryochloris*-like cells were also found inside the tunic of the larva isolated from *L. fragile*, indicating that the symbionts were transmitted to the progeny (Figure 5E inset). Vertical transmission of photosymbionts to larvae has often been observed in ascidians and is assumed to be essential for host survival [18], [78]. To date, three transmission modes have been described. The first mode involves the formation of a tunic extension at the posterior end of the larval trunk, just above the tail insertion called *rastrum* [40]. The *rastrum* has been described in most *Diplosoma* species with photosymbionts in the cloacal cavities [22], [40], [68], [78], [79], [80], [81], [82], [83], [84]. The second mode has been observed in some *Didemnum, Trididemnum* and *Lissoclinum* species and is associated with the adhesion of the photosymbionts to either the posterior end of the larval trunk or around the entire larva except for the sensory and adhesive organs [19], [21], [40], [78], [85], [86]. These ascidian species also harbor their photosymbionts in the cloacal cavities and, as in the first mode, symbionts are acquired when larvae pass through these cloacal cavities.

A more recently described transmission mode applies to ascidian species that harbor photosymbionts within their tunic. This process was described for *Trididemnum miniatum* and is thought to involve host cells acting as a vehicle for transporting symbionts from the tunic of the adult to that of the larvae [20]. Although no larvae were obtained from *T. solidum* and *T. cyanophorum*, the process described for *T. miniatum* or a similar mechanism may apply to the *Trididemnum* species analyzed in the present study. Our observations for *L. fragile* suggest that this species may also acquire their symbionts by an active transport mechanism during the formation of the deeper layer of the tunic, or inner tunic. In support of this hypothesis, the cyanobacteria were observed in the inner tunic separated by a folded cuticle from the outer tunic (Figure 5E inset). Photosymbionts are sufficiently abundant in the inner tunic to confer obvious pigment to the tadpole larve, except for the regions around the adhesive and sensory organs. Further research is needed to assess the exact process involved in the transfer of photosymbionts to the larvae and to determine whether ascidian cells are implicated in this process.

### Chlorophyll *d* in *Lissoclinum* aff. *fragile*

**Figure 6:**
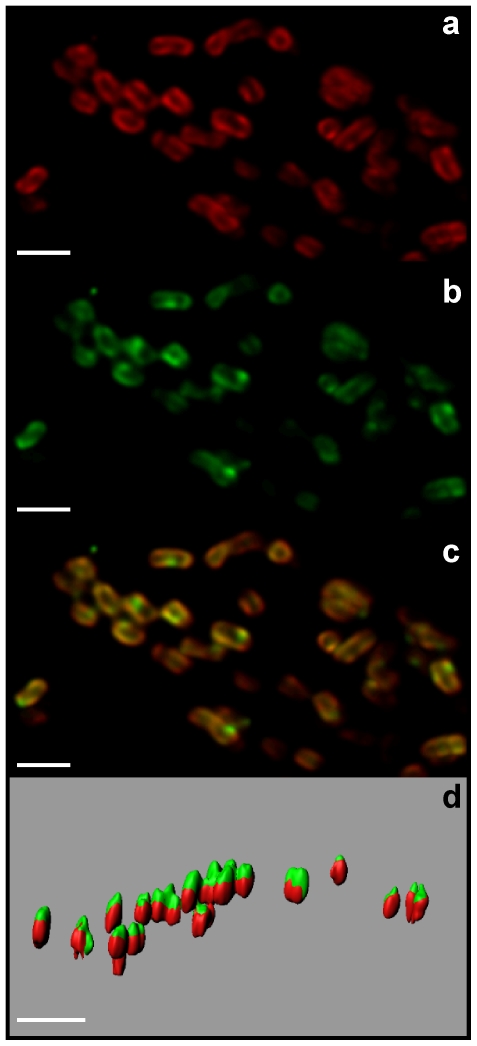
Confocal microscopical images and model reconstruction of *Acaryochloris*-like cells in *Lissoclinum* aff. *fragile*. Cells were excited with a 635 nm red laser and fluorescence was detected between 700–750 nm for chlorophyll *d* (a) and 640–670 nm for phycobiliproteins (b). (c) Composite image from combining the chlorophyll *d* (red) and the phycobiliproteins (green) channels. (d) 3D surface rendering after image deconvolution showing the emplacement of chlorophyll *d* and phycobiliproteins within the *Acaryochloris*-like cells. Scale bar = 2 µm.

Confocal microscopic examination revealed the presence of numerous cells containing both chlorophyll *d* (chl *d*) and phycobiliproteins (PBPs) in the tunic of *Lissoclinum* aff. *fragile* (Figure 6). Chl *d* is a predominant photosynthetic pigment in *Acaryochloris* species, a genus discovered to live in close association with ascidians [8], [17], [72], [73], [87]. Measurements performed on the deconvolved image stacks of these *Acaryochloris*-like cells revealed an average length of 1.75 µm (±0.38; SD), width of 1.13 µm (±0.24; SD), and volume of 2.32 µm^3^ (±1.07; SD). The diameter and length of the observed cells were within the range reported in the literature for *Acaryochloris* spp. (1.5–1.7 µm diameter and 1.8–2.1 µm in length [75], 1–1.5 µm in diameter and 1.5–3 µm in length [15]).

To date, *Acaryochloris* spp. have only been found growing on biofilms beneath didemnid ascidians [17], [72], [73] or forming small aggregations at the base of the ascidian tunic [8]. However, in this study, *Acaryochloris*-like cells were abundant (Figure 6) and appeared scattered through the tunic. Three-dimensional reconstruction of these cells also revealed that chl *d* and PBPs co-localized in the center of each *Acaryochloris*-like cell, however, they were compartmentalized, with chl *d* mainly present on one side and PBPs on the other (Figure 6d). A particular orientation of chl *d* and PBPs within the photosymbiont cell and the host tunic may be necessary to ensure optimal sunlight absorption.

Taken together with phylogentic analyses and TEM observations, our results indicate that *L. fragile* harbors photosymbionts from the *Acaryochloris* group within its tunic. Based on unique 16S rRNA and 16S–23S ITS sequences, we propose here to name *Candidatus Acaryochloris bahamiensis* nov. sp. for this photosymbiont. The importance of *A. bahamiensis* nov. sp. to the survival of its host remains to be assessed. However, vertical transfer of *A. bahamiensis* nov. sp. to the larvae suggests a close association between host and symbiont, and that this relationship is important to host survival. This is the first report of *Acaryochloris* forming a symbiotic relationship with an ascidian and living within its tunic.

### Conclusion

In conclusion, using molecular and electron microscopical techniques, we have shown that the ascidians examined in this study harbor a considerable diversity of photosymbionts. The primary taxa of symbionts found were *Synechocystis* in the tunic of *Trididemnum solidum* and *T. cyanophorum*, and *Acaryochloris*-like symbionts in *Lissoclinum fragile* and *L*. aff. *fragile*. Host identity strongly correlated with the identity of the photosymbionts found in the tunic, although in some cases (e.g. *Lissoclinum fragile*) differences could be related to sampling location. Analyses of 16S rRNA and 16S–23S ITS sequences from the symbionts in two varieties of *Lissoclinum fragile* revealed two major clades (F and J). Clade J could not be associated with any known cyanobacterium, while clade F was related to *Acaryochloris*. Ultrastructural examination confirmed the similarity of clade F symbionts with other species of *Acaryochloris*. These symbionts were also observed in the inner tunic of the larvae, suggesting that specialized mechanisms of vertical transmission exist. Analyses using CM revealed the presence of chl *d* and PBP, further reinforcing the classification of these photosymbionts as *Acaryochloris* spp. Based on these results and unique 16S rRNA and 16S–23S ITS sequences, we propose the name *Candidatus Acaryochloris bahamiensis* nov. sp. for this photosymbiont. Substantial research is still required to determine the diversity, host-specificity, and function of microbial symbionts in ascidians.
